# Lenalidomide plus rituximab Vs rituximab alone in relapsed or refractory indolent lymphoma: A cost‐effectiveness analysis

**DOI:** 10.1002/cam4.3121

**Published:** 2020-06-02

**Authors:** Peng‐Fei Zhang, Dan Xie, Feng Wen, Qiu Li

**Affiliations:** ^1^ Department of Medical Oncology Cancer Center West China Hospital Sichuan University Chengdu China; ^2^ West China Biomedical Big Data Center West China Hospital/West China School of Medicine Sichuan University Chengdu China; ^3^ Prenatal Diagnosis Center Department of Obstetrics and Gynecology West China Second University Hospital Sichuan University Chengdu China

**Keywords:** cost‐effectiveness, health economics, indolent lymphoma, lenalidomide, rituximab

## Abstract

**Background:**

The aim of the study was to evaluate the cost‐effectiveness of lenalidomide plus rituximab vs rituximab alone in patients with relapsed or refractory indolent lymphoma.

**Methods:**

A Markov decision model was established to carry out the cost‐effectiveness analysis. Three discrete health states, progression‐free survival (PFS), progressive disease (PD), and death, were included. Cycle length was set at 1 month, and utility scores were derived from previously published literature. The incremental cost‐effectiveness ratio (ICER) was defined as the primary endpoint, and the willingness‐to‐pay (WTP) threshold was set at $29,306.43 per quality‐adjusted life year (QALY). Both cost and effectiveness were determined using a 3% annual discount rate. Furthermore, one‐way and probabilistic sensitivity analyses were performed to check the robustness of the model.

**Results:**

Lenalidomide plus rituximab gained 6.08 QALYs at a cost of $120,979.62 while rituximab alone gained 4.84 QALYs at a cost of $48,052.11. The ICER of lenalidomide plus rituximab vs rituximab alone was $58,812.51/QALY. The parameters most significantly influenced the model were the utility values for the PFS state, the duration of the PFS state in the lenalidomide plus rituximab group, and the cost of lenalidomide. The probability of lenalidomide plus rituximab or rituximab alone being the most cost‐effective option was 0% and 100%, respectively, at a WTP threshold of $29,306.43/QALY.

**Conclusions:**

Lenalidomide plus rituximab is not a cost‐effective strategy compared with rituximab monotherapy for relapsed or refractory indolent lymphoma from a Chinese societal perspective.

## Graphical abstract

This is the first study to evaluate the cost‐effectiveness of lenalidomide plus rituximab vs rituximab alone in patients with relapsed or refractory indolent lymphoma. Lenalidomide plus rituximab is not a cost‐effective strategy compared with rituximab monotherapy for patients with relapsed or refractory indolent lymphoma from a Chinese societal perspective.

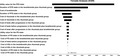

## 1 INTRODUCTION

Non‐Hodgkin lymphoma (NHL) represents a family of lymphoid neoplasms with different morphologic, immunophenotypic, genetic, and clinical features. It is estimated that approximately 400 thousand new cases of NHL occurred worldwide in 2012, which resulted in 199 700 deaths.^1^ Indolent lymphoma is a type of low‐grade NHL that tends to grow and spread slowly. Indolent lymphomas constitute approximately one‐third of NHL, and the most common types are follicular lymphoma (FL) and marginal zone lymphoma (MZL).^2^, ^3^ Indolent lymphomas often have high response rates to initial treatments; however, most patients relapse afterwards.^4^, ^5^ Chemotherapy plus anti‐CD20 monoclonal antibodies and other targeted agents, such as phosphatidylinositol 3‐kinase inhibitors, are considered the standard treatment options for patients with relapsed/refractory FL and MZL.^6^, ^7^, ^8^ However, chemotherapy drugs are always associated with a series of side effects, such as myelosuppression, immunosuppression, and cardiac toxic effects.^9^, ^10^ To reduce the incidences of side effects in the treatment of indolent lymphomas, rituximab monotherapy has been investigated in several studies and demonstrated to be effective for indolent lymphoma patients who had previously responded to rituximab.^11^, ^12^ Thus, rituximab monotherapy has been approved by the US Food and Drug Administration for the treatment of these patients.^13^

Lenalidomide, an immunomodulatory agent, can bind the cereblon E3 ubiquitin ligase complex and result in ubiquitination of transcription factors Aiolos and Ikaros, which can boost the apoptosis of tumor cells and the activation of T cells and natural killer (NK) cells.^14^, ^15^, ^16^ Recently, the efficacy and safety of lenalidomide combined with rituximab compared to placebo plus rituximab in patients with relapsed or refractory indolent NHL was investigated in the AUGMENT trial.^17^ Lenalidomide plus rituximab significantly prolonged progression‐free survival (PFS) compared to placebo plus rituximab (39.4 months (95% CI, 22.9 months to not reached) vs 14.1 months (95% CI, 11.4 to 16.7 months), hazard ratio (HR) 0.46 (95% CI, 0.34 to 0.62; *P* < .001)), suggesting that lenalidomide plus rituximab might be an effective treatment option for patients with relapsed or refractory indolent lymphoma.

Despite the significant efficacy achieved by the addition of lenalidomide, the high price of lenalidomide may weaken the benefit of the novel regimen and increase the cost of treatment for indolent lymphoma. Given the rapidly growing health‐care expenditures and limited health‐care resources worldwide, evaluating novel treatment options from other aspects, such as pharmacoeconomic profiles, is necessary.^18^, ^19^ The aim of this study was to investigate the pharmacoeconomic profile of lenalidomide plus rituximab vs rituximab alone in patients with relapsed or refractory indolent lymphoma from a Chinese societal perspective.

## 2 METHODS

### 2.1 Model structure

**Figure 1:**
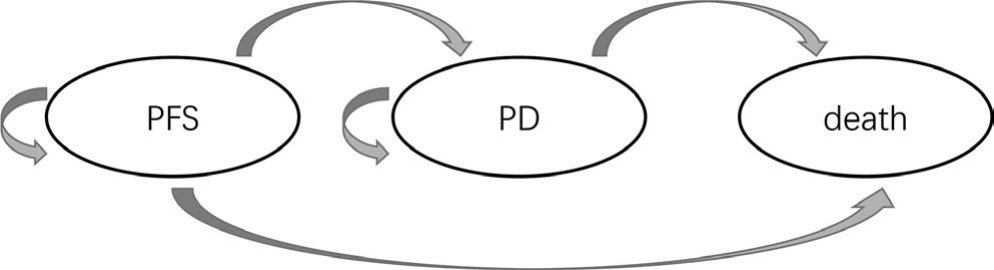
Markov model diagram for patients with relapsed or refractory indolent lymphoma. PFS, progression‐free survival; PD, progressive disease

In the current study, we carried out a cost‐effectiveness analysis comparing the cost and health benefits associated with lenalidomide plus rituximab with rituximab alone using a Markov decision model, which simulated the disease course of patients with relapsed or refractory indolent lymphoma over their lifetimes. Three discrete health states, PFS, progressive disease (PD), and death, were included in the model. The baseline characteristics of the patients in the hypothetical cohorts (1000 patients for each group) were assumed to be consistent with those of the AUGMENT trial, and all patients were assumed to enter the model in the PFS state. At the end of each Markov cycle, one patient would stay within the state at the beginning, or change to another state as described in Figure 1. The cycle length and lifetime horizon in the model were set to 1 month and 10 years, respectively. Half‐cycle correction was used for the cyclical transitions to adjust for the timing of the transition between health states. The incremental cost‐effectiveness ratio (ICER) was defined as the primary endpoint of the analysis, and the willingness‐to‐pay (WTP) threshold was set at $29,306.43/QALY (3 × per capita GDP of China, 2018) according to the WHO guideline for cost‐effectiveness analysis.^20^ The analysis was performed from the perspective of Chinese society, and both cost and effectiveness were determined using a 3% annual discount rate.

### 2.2 Efficacy input and utility

Efficacy data in the model were derived from the AUGMENT trial, in which a total of 358 patients were randomly assigned to the lenalidomide plus rituximab group (n = 178) or the placebo plus rituximab group (n = 180), and the median follow‐up was 28.3 months at the final analysis (Table 1). As individual patient data were not available, survival data were extracted from the survival curves using a plot digitizer software (DigitizeIt, version 2.0, www.digitizeit.de); then, Weibull survival models were used to fit the Kaplan‐Meier curves for PFS and overall survival (OS) derived from the AUGMENT trial (Figure S‐1).^21^ Fitting parameters (Akaike's Information Criteria (AIC), Bayesian Information Criterion (BIC), the estimated Weibull scale (*λ*), and shape (*γ*) parameters, *R* ^2^) are presented in Table 2 and Table S1. In the current analysis, the median values of survival were used to estimate monthly transition probabilities in both study arms using the following formula: Risk for an event (1 month) = [1−(0.5) (1/median time to event)], which was derived from the following equations: *P* = 1 − e^−R^ and R = − ln[0.5]/(time to event/number of treatment cycles). Quality‐adjusted life year (QALY) was regarded as the primary effectiveness result. Utility values ranged from 0.0 to 1.0 (where 1.0 represented perfect health and 0.0 represented death). Utility values for the modeled health states and disutility values for adverse events (AEs) in the study are listed in Table 2 and were derived from previously published literature.^22^, ^23^

**Table 1:** Efficacy and safety data derived from the AUGMENT trial

	Lenalidomide plus rituximab	Rituximab
Survival, median (95% CI)		
mOS (months)	—	—
mPFS (months)	39.4 (22.9‐NR)	14.1 (11.4‐16.7)
Grade 3‐4 AEs (%)		
Neutropenia	50	13
Diarrhea	3	0
Cough	1	0
Fatigue	1	1
Pyrexia	1	2
Leukopenia	7	2
Upper respiratory tract infection	1	2
Anemia	5	1
Headache	1	0
Infusion‐related reaction	2	0
Thrombocytopenia	2	1
Asthenia	1	1
Decreased appetite	1	0
Muscle spasms	1	1
Abdominal pain	1	0
Pruritus	1	0
Dyspnea	1	1
Rash	1	1
Tumor flare	1	0
Alanine aminotransferase increased	2	1
Influenza	1	0

Abbreviations: AEs, adverse events; CI, confidence interval; mOS, median overall survival; mPFS, median progression‐free survival.

**Table 2:** Parameters used in the model

Parameters	Value	Range	References
Weibull parameters of PFS for lenalidomide plus rituximab arm			
Scale (*λ*)	0.008483	Fixed	[17]
Shape (*γ*)	1.359419	Fixed	[17]
AIC	650.4936	Fixed	[17]
BIC	656.8346	Fixed	[17]
Weibull parameters of OS for lenalidomide plus rituximab arm			
Scale (*λ*)	0.001519	Fixed	[17]
Shape (*γ*)	1.243833	Fixed	[17]
AIC	201.0573	Fixed	[17]
BIC	207.4321	Fixed	[17]
Weibull parameters of PFS for rituximab arm			
Scale (*λ*)	0.036832	Fixed	[17]
Shape (*γ*)	1.030835	Fixed	[17]
AIC	878.593	Fixed	[17]
BIC	885.0119	Fixed	[17]
Weibull parameters of OS for rituximab arm			
Scale (*λ*)	0.001581	Fixed	[17]
Shape (*γ*)	1.383142	Fixed	[17]
AIC	295.9283	Fixed	[17]
BIC	302.1881	Fixed	[17]
Utility/Disutility values			
PFS	0.859	0.687‐1	[22]
PD	0.798	0.638‐0.958	[22]
Death	0	0‐0	[22]
Neutropenia	−0.131	−(0.105‐0.157)	[23]
Leukopenia	−0.131	−(0.105‐0.157)	[23]
Anemia	−0.119	−(0.095‐0.143)	[23]

Abbreviations: AIC, Akaike's Information Criteria; BIC, Bayesian Information Criterion; OS, overall survival; PD, progressive disease PFS, progression‐free survival.

### 2.3 Cost input

Cost was estimated from a Chinese societal perspective in the study and only direct cost was calculated. The following components were included: cost of the drugs, cost of the laboratory tests and radiological examination, cost of grade 3‐4 AE‐related treatment, and cost of treatments in the PD state. The dosages of lenalidomide and rituximab were derived from the AUGMENT trial; the lenalidomide plus rituximab dosing included 20‐mg oral lenalidomide daily on days 1 to 21 plus intravenous rituximab 375 mg/m^2^ on days 1, 8, 15, and 22 of cycle 1 and on day 1 of cycles 2 to 5 every 28 days. Rituximab alone was administered similarly, and the dosages of rituximab were calculated based on the normal body height and weight (a weight of 65 kg and a height of 1.64 m, body surface area with a mean value of 1.72 m^2^).^24^ As data on post‐progression treatments were not reported in the AUGMENT trial, trial‐specific cost for PD state could not be derived. Thus, the cost estimated for further treatments after disease progression was based on an established practice pattern for relapsed FL in a previous study.^25^ Meanwhile, the cost of grade 3‐4 AEs was estimated based on published guidelines and our clinical coauthors’ expert opinion in the management strategies for each AE, which was obtained by multiplying the incidence and the unit cost for each type of AE.^26^, ^27^ Unit costs were derived from data of the local health system or the National Development and Reform Commission (NDRC) of China. The cost of supportive care was not included in the study, as these treatments are complex and heterogeneous. All costs were converted into US dollars based on the exchange rate in 2018 (1 USD = 6.6174 CNY).

### 2.4 Sensitivity analyses

To check the robustness of the model, one‐way sensitivity analyses were performed with several key parameters, such as the duration of the PFS state and the costs of lenalidomide and rituximab. Parameters ranged between ± 20% in the one‐way sensitivity analyses, the results of which are presented as tornado diagrams. Meanwhile, probabilistic sensitivity analyses were also performed using a Monte Carlo simulation. Different distributions were fitted for each parameter (gamma distribution for cost parameters, exponential distribution for survival parameters, and triangle distribution for health parameters). Based on these distributions, 1000 iterations of 1000 simulated patients were modeled, and the results of the probabilistic sensitivity analysis were presented as probabilistic sensitivity acceptability curves and scatterplots.

The R software package (version 3.6.1; R Development Core Team, Vienna, Austria) and TreeAge 2011 (TreeAge) were used for model creation and data analysis.

## 3 RESULTS

### 3.1 Base case analysis

Over a lifetime horizon of 10 years, the effectiveness gained by the lenalidomide plus rituximab group was 6.08 QALYs, while the effectiveness of rituximab alone was 4.84 QALYs. Meanwhile, the costs of the lenalidomide plus rituximab group and rituximab alone group were $120,979.62 and $48,052.11, respectively (Table 3). The ICER of the lenalidomide plus rituximab group vs the rituximab alone group was $58,812.51/QALY, indicating that lenalidomide plus rituximab is unlikely to be cost‐effective compared with rituximab alone based on the WTP threshold of $29,306.43/QALY.

**Table 3:** Base case analysis of the decision model

	Lenalidomide plus rituximab	Rituximab
Monthly cost ($)		
Cost of lenalidomide	5496.42	—
Cost of rituximab	1571.22	1571.22
Cost of tests	144.29	144.29
Cost of grade 3‐4 AEs	19.44	7.01
Cost of treatments after PD	886.68	1292.68
Cost of tests after PD	144.29	144.29
Lifetime cost ($)		
PFS state	108 667.91	31 503.56
PD state	12 311.71	16 548.54
Total cost	120 979.62	48 052.11
Incremental cost	72 927.51	
Effectiveness (QALYs)		
Effectiveness for the PFS state	3.05	1.48
Effectiveness for the PD state	3.03	3.36
Total effectiveness	6.08	4.84
Incremental effectiveness	1.24	
ICER	58 812.51	

Abbreviations: AEs, adverse events; BSC, best‐supportive care; ICER, incremental cost‐effectiveness ratio; QALYs, quality‐adjusted life years; PD, progressive disease; PFS, progression‐free survival.

### 3.2 One‐way sensitivity analyses

**Figure 2:**
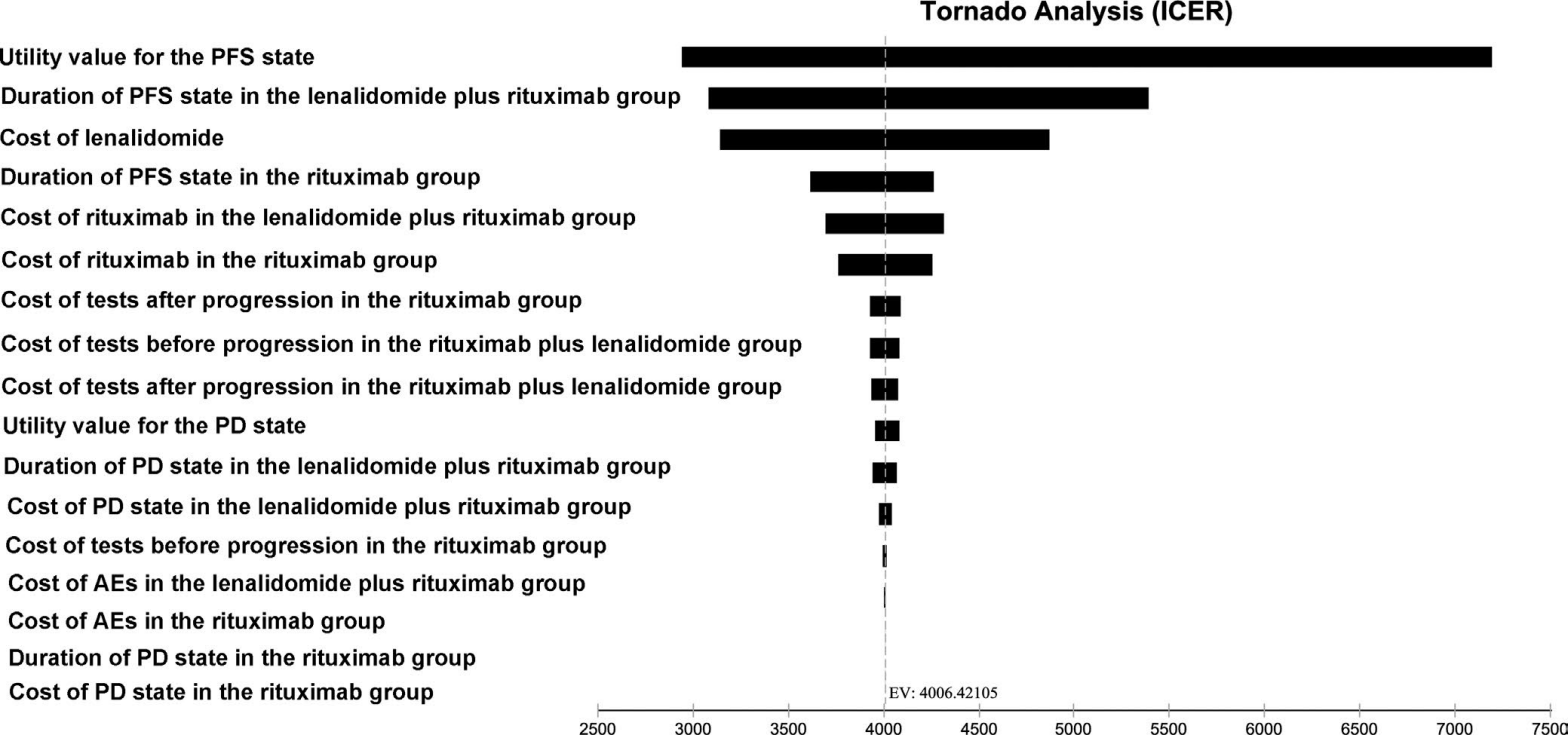
Tornado diagram of the one‐way sensitivity analysis for ICER. PFS, progression‐free survival; PD, progressive disease; AEs, adverse events; ICER, incremental cost‐effectiveness ratio

One‐way sensitivity analyses were performed in the study to investigate the impact of key variables on the results. As presented in Figure 2, the model was most sensitive to the utility values for the PFS state, the duration of the PFS state in the lenalidomide plus rituximab group, and the cost of lenalidomide. In addition, the model was also sensitive to the duration of the PFS state in the rituximab group and the cost of rituximab. On the other hand, other variables, such as the cost of tests and cost of AEs, had little impact on the results of the model.

### 3.3 Probabilistic sensitivity analyses

**Figure 3:**
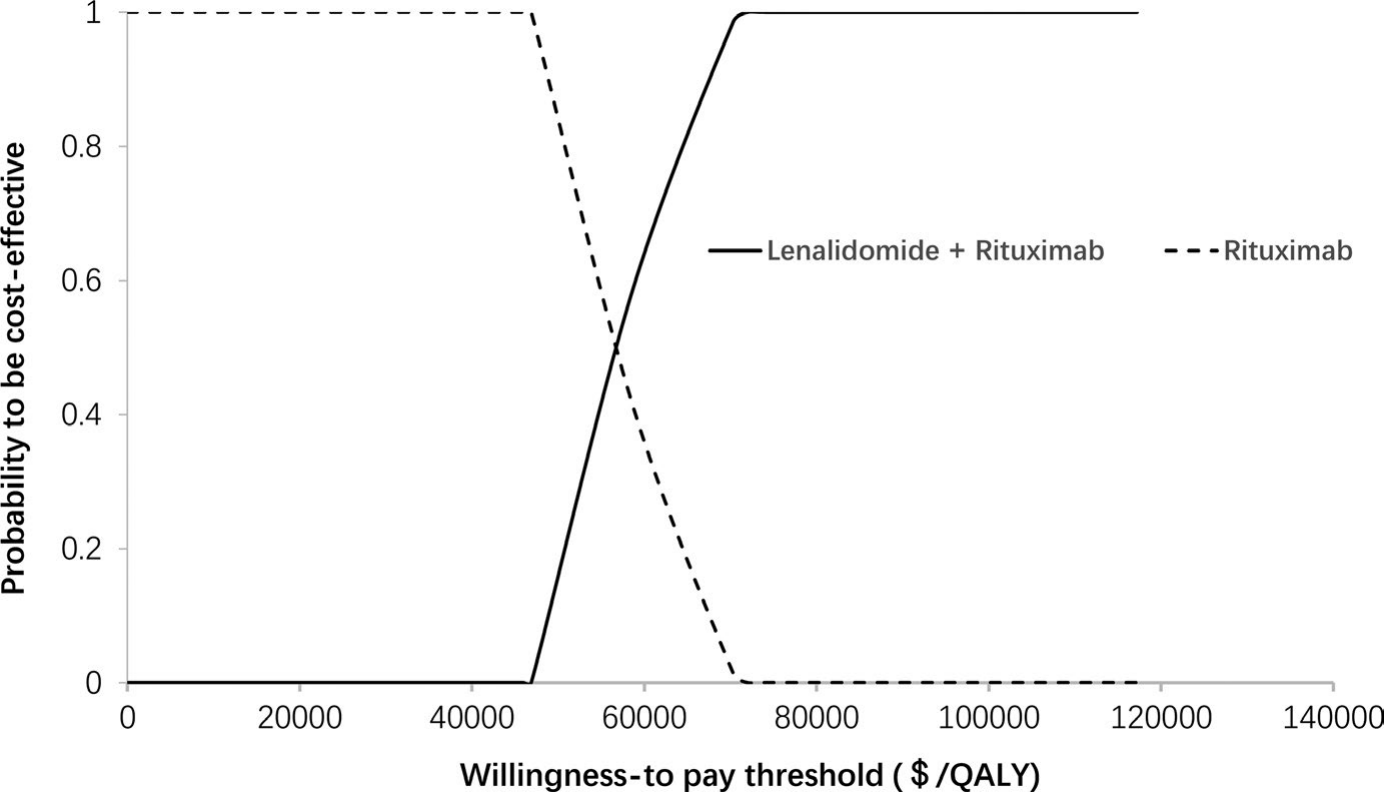
Cost‐effectiveness probabilistic acceptability curves. CE, cost‐effectiveness; QALY, quality‐adjusted life year

**Figure 4:**
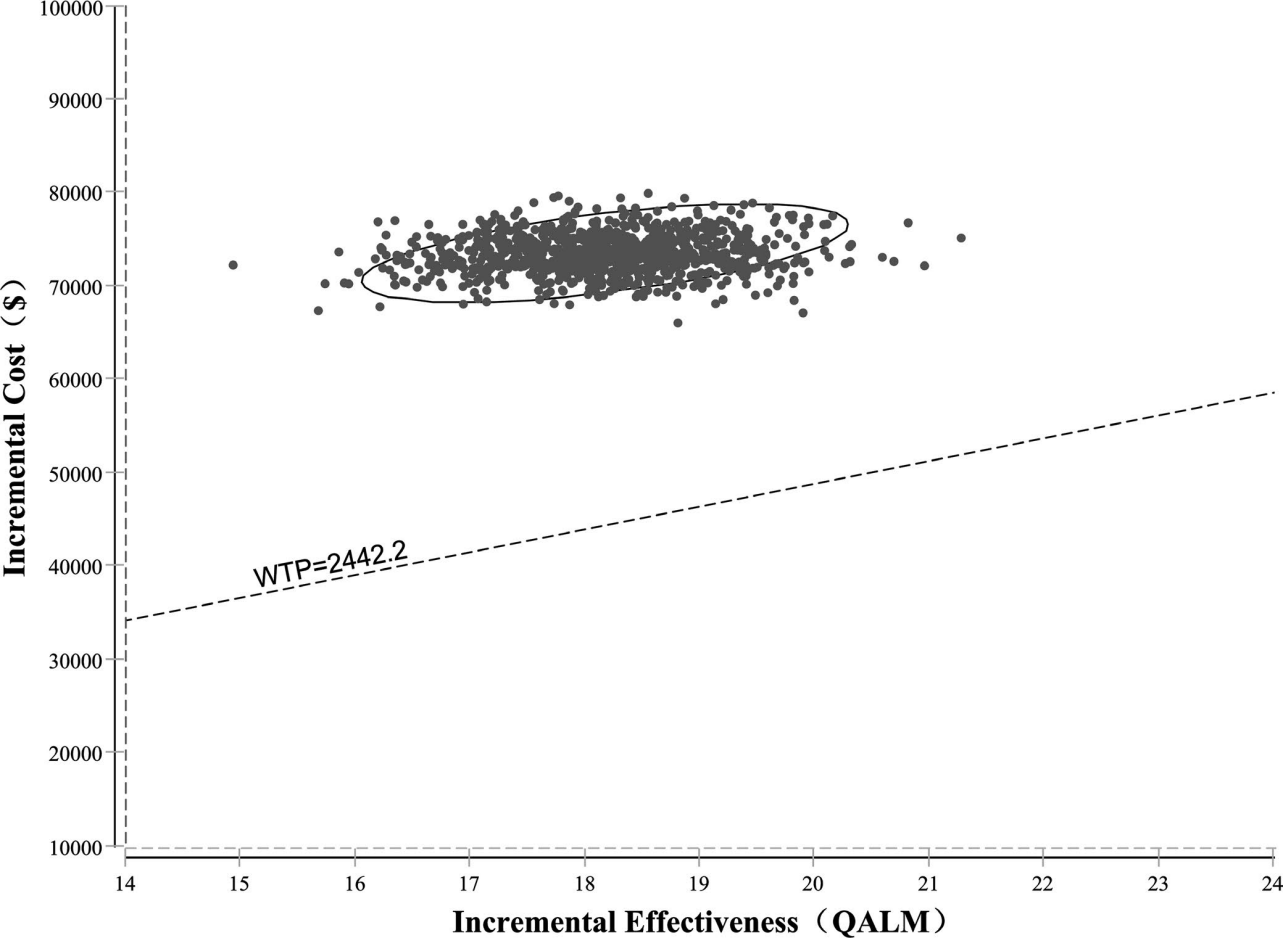
Scatter plots diagrams of lenalidomide plus rituximab vs rituximab monotherapy. QALM, quality‐adjusted life month

We also performed probabilistic sensitivity analyses based on a Monte Carlo simulation of 1000 simulations. At the WTP threshold of $29,306.43/QALY, the probability of rituximab alone or lenalidomide plus rituximab to be cost‐effective was 0% and 100%, respectively (Figure 3). Moreover, the incremental cost‐effectiveness scatterplots showed that all scatter points were above the WTP threshold line, which also indicated that lenalidomide plus rituximab was not the dominant option compared with rituximab alone in the treatment of patients with relapsed or refractory indolent lymphoma at the WTP threshold of $29,306.43/QALY (Figure 4).

## 4 DISCUSSION

FL and MZL are the most common types of indolent lymphoma, and rituximab monotherapy has been applied in the treatment of patients with relapsed/refractory FL and MZL for avoiding severe side effects. Recently, investigators compared lenalidomide plus rituximab with rituximab monotherapy in the treatment of patients with relapsed or refractory indolent NHL in the AUGMENT trial. Lenalidomide plus rituximab was demonstrated to significantly prolong PFS compared to placebo plus rituximab, indicating that lenalidomide plus rituximab was an effective option for patients with relapsed or refractory indolent lymphoma. Although the study met its primary end point, the high price of lenalidomide may counterbalance its survival effect. Thus, in this study, we evaluated the cost‐effectiveness of lenalidomide plus rituximab vs rituximab alone for patients with relapsed or refractory indolent lymphoma from a Chinese societal perspective. Lenalidomide plus rituximab increased the effectiveness by 1.24 QALYs compared with rituximab monotherapy. However, the lenalidomide plus rituximab group also incurred a higher cost. The costs of lenalidomide plus rituximab and rituximab monotherapy were $120,979.62 and $48,052.11, respectively. Overall, the ICER of the two groups (lenalidomide plus rituximab vs rituximab alone) was $58,812.51/QALYs, suggesting rituximab was the dominant option compared with lenalidomide plus rituximab for patients with relapsed or refractory indolent lymphoma at the WTP threshold of $29,306.43/QALY from a Chinese societal perspective.

In the sensitivity analyses, the utility values for the PFS state, the duration of the PFS state in the lenalidomide plus rituximab group, and the cost of lenalidomide were key parameters that significantly influenced the ICER. As reported in the AUGMENT trial, the lenalidomide plus rituximab group had a significantly longer PFS than the placebo plus rituximab group (39.4 months (95% CI, 22.9 months to not reached) vs 14.1 months (95% CI, 11.4‐16.7 months)), which contributed greatly to the incremental effectiveness of the former treatment. However, the effectiveness of the PD state between the two groups was not significantly different based on the Weibull survival models and Markov models (3.03 QALYs in the lenalidomide plus rituximab group and 3.35 in the rituximab group). Thus, the PFS in the two groups could significantly influence the results of the model. Meanwhile, besides the duration of the PFS state, the quality of life might be another key parameter in determining effectiveness. As expected, the results of the one‐way sensitivity analyses showed that the utility values for the PFS state significantly influenced the model. In addition, the costs of lenalidomide and rituximab were two other parameters that significantly influenced the model. Lenalidomide and rituximab are substantially expensive, and there is no doubt that these two parameters could greatly impact the results of the model. Decreasing the prices of the two drugs might be a solution to improve the pharmacoeconomic profile of lenalidomide plus rituximab vs rituximab in the treatment of patients with relapsed or refractory indolent lymphoma.

Current treatment options for relapsed or refractory indolent lymphoma include rituximab monotherapy, bendamustine, or other chemotherapy with or without obinutuzumab or rituximab.^6^, ^7^, ^8^ As health expenditures have become one of the most severe issues worldwide, especially China, which has limited health resources and a large population, investigating the cost‐effectiveness of novel strategies has become important. Soini *et al* evaluated the cost‐effectiveness of RCHOP, RCHOP‐R, and CHOP in the treatment of patients with relapsed or refractory indolent lymphoma based on data from the EORTC20981 trial.^28^ The ICER values were €18,147/QALY for RCHOP‐R vs RCHOP, €14,360/QALY for RCHOP‐R vs CHOP, and €12,123/QALY for RCHOP vs CHOP, suggesting that RCHOP‐R was the optimal option at a WTP of €18,399/QALY. In another study, Blommestein *et al* investigated the cost‐effectiveness of rituximab maintenance vs observation in relapsed or refractory FL patients who responded to second‐line chemotherapy based on data from the EORTC20981 trial, the Netherlands Cancer Registry, and two population‐based registries.^22^ Despite the differences in real‐world and trial populations, rituximab maintenance was demonstrated to be cost‐effective using real‐world data as well as results from long‐term trial follow‐up. In this study, we first reported the cost‐effectiveness of lenalidomide plus rituximab compared with rituximab alone for patients with relapsed or refractory indolent lymphoma. In addition to efficacy and safety data, the study could yield additional pharmacoeconomic data for the two treatment options, which could provide more useful evidence for doctors and patients to select the optimal treatment options.

Some limitations should be addressed in our study. First, the utility scores for the three health states were derived from previously published studies, which may not reflect the true situation for Chinese patients. Second, although data on AEs were reported in the AUGMENT trial, an accurate estimation of the cost of AEs is difficult. In the analysis, only grade 3 to 4 AEs were included. Fortunately, the cost of AEs had a minor influence on the ICER based on the one‐way sensitivity analyses, which may decrease the influence of the estimation of the cost of AEs on the results of the study. Third, data on treatments for PD states were not reported in the AUGMENT trial and the cost estimated for further treatments for PD states was based on previous study, which may also decrease the robustness of our analysis. Fourth, despite the merits of the study, we merely investigated the cost‐effectiveness of lenalidomide plus rituximab compared with that of rituximab alone for patients with relapsed or refractory indolent lymphoma and did not include other treatment options in the study, as there are no head‐to‐head trials that have compared the effect of these regimens with treatment regimens in the AUGMENT study. Thus, head‐to‐head trials comparing the efficacy and safety of lenalidomide plus rituximab with other standard treatment regimens are urgently needed.

## 5 CONCLUSION

In conclusion, we evaluated the cost‐effectiveness of lenalidomide plus rituximab vs rituximab alone for patients with relapsed or refractory indolent lymphoma from a Chinese societal perspective, and demonstrated that lenalidomide plus rituximab is not a cost‐effective regimen compared with rituximab alone. The results of the study could provide evidence of pharmacoeconomic profiles other than the efficacy and safety provided by the AUGMENT trial for decision‐ and policy makers.

## CONFLICT OF INTEREST

None declared.

## AUTHORS CONTRIBUTION

Peng‐Fei Zhang: Conceptualization, Methodology, Data curation, Formal analysis, Investigation, Validation, and Writing (original draft, review, and editing). Dan Xie: Data curation, Formal analysis, Investigation, Validation, and Writing (original draft, review, and editing). Feng Wen: Data curation, Formal analysis, Investigation, Validation, and Writing (review and editing). Qiu Li: Conceptualization, Methodology, Funding acquisition, Investigation, Validation, and Writing (review and editing).

## Supporting information

Fig S1

Table S1

## Data Availability

The data that support the findings of this study are available from the corresponding author upon reasonable request. This work is under Creative Common CC‐B. The data used in the current study are available from the corresponding author upon reasonable request.
