# Supplementary figures and images for: Lenalidomide plus rituximab Vs rituximab alone in relapsed or refractory indolent lymphoma: A cost‐effectiveness analysis
Source: Cancer Med. 2020 Jun 2;9(15):5312–9. doi: 10.1002/cam4.3121 (PMC7402838; doi:10.1002/cam4.3121)

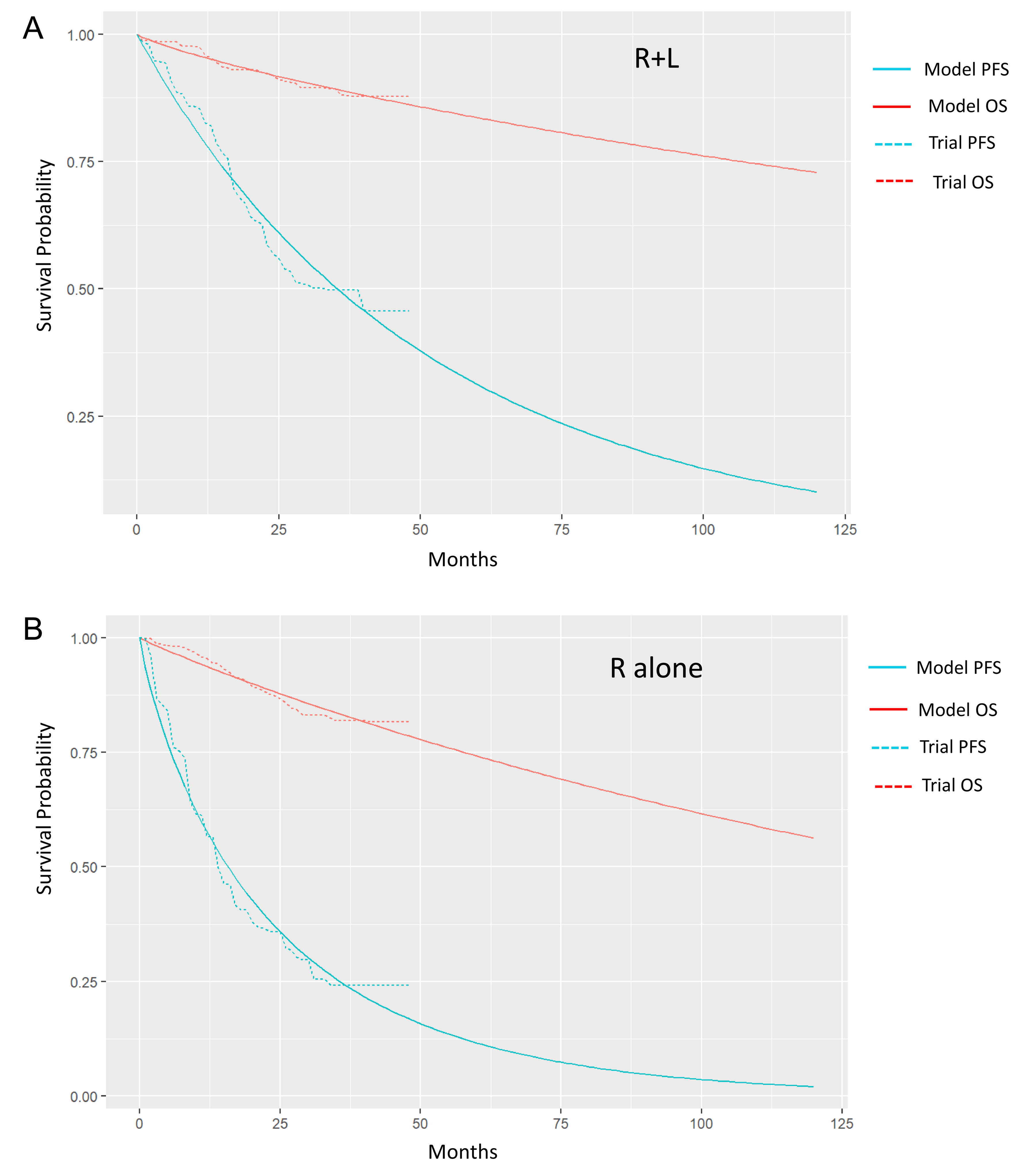

Supplement: Supplementary file 1 — Fig S1 [file CAM4-9-5312-s001.tif]
