# Supplementary material for: Lenalidomide plus rituximab Vs rituximab alone in relapsed or refractory indolent lymphoma: A cost‐effectiveness analysis
Source: Cancer Med. 2020 Jun 2;9(15):5312–9. doi: 10.1002/cam4.3121 (PMC7402838; doi:10.1002/cam4.3121)
Supplement: Supplementary file 2 — Table S1 [file CAM4-9-5312-s002.docx]

**Table S-1 Parameters for Weibull distribution and Exponential distribution model.**

| **Parameters** | **Value** |
| --- | --- |
| **R^2^ for Weibull model of PFS in lenalidomide plus rituximab arm** | 0.9842048 |
| **R^2^ for Weibull model of OS in lenalidomide plus rituximab arm** | 0.9644299 |
| **R^2^ for Weibull model of PFS in rituximab alone arm** | 0.9721409 |
| **R^2^ for Weibull model of OS in rituximab alone arm** | 0.9490481 |
| **R^2^ for Exponential distribution model of PFS in lenalidomide plus rituximab arm** | 0.9633411 |
| **R^2^ for Exponential distribution model of OS in lenalidomide plus rituximab arm** | 0.9463877 |
| **R^2^ for Exponential distribution model of PFS in rituximab alone arm** | 0.9585869 |
| **R^2^ for Exponential distribution model of OS in rituximab alone arm** | 0.9476488 |

PFS, progression-free survival; OS, overall survival
